# Significance of S100B Protein as a Rapid Diagnostic Tool in Emergency Departments for Traumatic Brain Injury Patients

**DOI:** 10.3390/jpm13121724

**Published:** 2023-12-18

**Authors:** Rakesh Jalali, Izabela Godlewska, Magdalena Fadrowska-Szleper, Agata Pypkowska, Adam Kern, Jacek Bil, Joanna Manta, Jerzy Romaszko

**Affiliations:** 1 Department of Emergency Medicine, School of Medicine, Collegium Medicum, University of Warmia and Mazury, 10-082 Olsztyn, Poland; iza.gdlwsk@gmail.com (I.G.); magdafadrowska@gmail.com (M.F.-S.); pypkowska.agata@gmail.com (A.P.);; 2 Clinical Emergency Department, Regional Specialist Hospital, 10-561 Olsztyn, Poland; 3 Department of Cardiology and Internal Medicine, School of Medicine, Collegium Medicum, University of Warmia and Mazury, 10-082 Olsztyn, Poland; adam.kern@uwm.edu.pl; 4 Department of Invasive Cardiology, Centre of Postgraduate Medical Education, 01-813 Warsaw, Poland; jacek.bil@cmkp.edu.pl; 5 Department of Family Medicine and Infectious Diseases, School of Medicine, Collegium Medicum, University of Warmia and Mazury, 10-082 Olsztyn, Poland; jerzy.romaszko@uwm.edu.pl

**Keywords:** emergency department, traumatic brain injury (TBI), S100B protein, alcohol, diagnostic process

## Abstract

Traumatic brain injuries (TBIs) are not only the leading cause of death among people below 44 years of age, but also one of the biggest diagnostic challenges in the emergency set up. We believe that the use of serum biomarkers in diagnosis can help to improve patient care in TBI. One of them is the S100B protein, which is currently proposed as a promising diagnostic tool for TBI and its consequences. In our study, we analyzed serum biomarker S100B in 136 patients admitted to the Emergency Department of the Regional Specialist Hospital in Olsztyn. Participants were divided into three groups: patients with head trauma and alcohol intoxication, patients with head trauma with no alcohol intoxication and a control group of patients with no trauma or with injury in locations other than the head. In our study, as compared to the control group, patients with TBI had a significantly higher S100B level (both with and without intoxication). Moreover, in both groups, the mean S100B protein level was significantly higher in patients with pathological changes in CT. According to our study results, the S100B protein is a promising diagnostic tool, and we propose including its evaluation in routine regimens in patients with TBI.

## 1. Introduction

Injuries are the leading cause of death among people below 44 years of age [1]. According to the CDC (Centers for Disease Control and Prevention), more than 82,700 traumatic brain injury (TBI)-related hospitalizations were recorded in the USA in 2001, and the number almost tripled in 2020 [2,3]. However, deaths are only the tip of the iceberg. Among 223,135 TBI incidents recorded in the USA in 2019, it was the cause of death of 60,611 patients, whereas thousands of people became disabled [3]. TBIs lead to severe socio-economic consequences via reducing the quality of life, not only of patients but also their families, and generating costs of billions of dollars annually [4]. According to Brazinova et al., the TBI incidence rate in Europe ranges between 47.3 (Spain) and 849 (Italy) per 100,000 people per year [5].

It is possible to identify specific groups with a higher risk of TBI. For example, people over 75 years of age have the highest risk of hospitalization and death related to TBI [3]. TBI is more frequent among homeless people and marginally housed individuals due to alcohol and drug dependence, mental health problems, suicide attempts, and sociological problems [6,7]. TBI usually results from direct trauma or acceleration–deceleration forces in road traffic accidents or violence. Brain tissue is vulnerable to the effects of trauma because of its high metabolism and low ability to function without a continuous energy supply.

TBIs can be classified into focal brain injuries and diffuse injuries. The first type can be identified in diagnostic imaging; therefore, basic diagnostic tools are used in emergency departments (EDs), especially computed tomography (CT). Nevertheless, indiscriminate CT use generates excessive costs and increases the risk of radiation exposure. Studies have shown that a third of malignant tumors found in patients with CT performed between 35 and 54 years of age result from this procedure [8]. Considering repeatedly occurring mild TBIs in groups of patients with a tendency to fall, such as elderly patients, alcohol addicts, or homeless people, precluding unnecessary CT scanning can significantly reduce its side effects. National Institute for Health and Care Excellence (NICE) Guidelines scales are one of the tools made for assessing the utility of obtaining CT scans and are used worldwide [9]. According to NICE Guidelines, the need for diagnostic imaging should be assessed mainly based on patients’ clinical state, Glasgow Coma Scale (GCS) score, age, mechanism of injury, and medication. Moreover, it has been reported that CT imaging shows negative results in the vast majority of mild TBI cases with patients who have a GCS score between 13 and 15 [10].

When dealing with TBI, not only is treating the primary brain damage important, but the prevention of secondary brain damage is also essential. Although the exact mechanisms of brain tissue response to stress and injury are yet to be discovered, we believe that it is possible to establish diagnostic tools with the employment of serum protein biomarkers. Many serum proteins such as glial fibrillary acidic protein, ubiquitin C-terminal hydrolase L1, neuron-specific enolase, and S100B are under investigation for their possible role as a marker for TBI (reviewed in [11]). Among these serum proteins, S100B has been extensively investigated for its use as a biomarker for TBI [12,13]. Its automated assays are readily available and easy to use.

Establishing a new diagnostic standard that would include TBI biomarkers could not only prevent unnecessary radiation in some groups of patients and thus its side effects but also help to estimate the possible injury outcome. The use of serum biomarkers in diagnostics can help to improve care in TBI in two ways. These markers can be a useful tool in assessing the necessity for diagnostic imaging and estimating the extensiveness of injury, which, consequently, can contribute to choosing the right therapeutic pathway.

In our study, we analyzed serum biomarker S100B, a calcium-binding protein mainly found in the astroglia and Schwann cells of the central nervous system (CNS). The aim of this study was to evaluate a correlation between CT with evidence of brain injury (positive CT) and serum S100B and assess the possible use of the S100B protein to determine a need for CT in two groups of patients: with and without alcohol intoxication at the time of injury.

Our study focused on alcohol-intoxicated patients, a group often admitted to the ED due to TBI. Furthermore, we wanted to determine whether alcohol intoxication affected the S100B serum level. The S100B protein test could help choose the right treatment pathway and verify the severity of the injury. Moreover, collecting information from and evaluating the clinical state of alcohol-intoxicated patients is difficult, making it harder to assess risk factors for severe injury (for example, mechanism of injury) and CT scan necessity.

## 2. Materials and Methods

The present observational study, approved by the local ethical committee of the University of Warmia and Mazury (approval reference number 21/2016 dated 18 May 2016), was based on a retrospective analysis of medical records of patients hospitalized in the Clinical Emergency Department of the Regional Specialist Hospital in Olsztyn, Poland. Patients’ medical records were provided to researchers with names and surnames being substituted for codes (anonymized), and all the data collection methods were in compliance with the Helsinki Declaration.

One hundred thirty-six patients admitted to the ED of the Regional Specialist Hospital between 2016 and 2018 in Olsztyn were included in this study. Participants were divided into three groups: patients with head trauma and alcohol intoxication (TrAlc; *n* = 49), patients with head trauma with no alcohol intoxication (NonTrAlc; *n* = 58), and the control group, which included patients with no trauma or with injury in locations other than the head (*n* = 29). Alcohol intoxication was defined as a blood alcohol concentration ≥ 50 mg/dL. We adopted the definition of TBI according to the NICE guidelines where, mild, moderate, and severe TBI are defined by the GCS ranges of 13–15, 9–12, and 8 or less, respectively [14].

Exclusion criteria were as follows: age under 18, neurosurgical intervention in past medical history, brain tumor, severe hypoxia, carbon monoxide intoxication, body temperature over 38.5 °C, malignant melanoma, and cardiac arrest caused by brain injury.

On admission, serum alcohol and S100B level was tested in all groups. CT of the head was performed in the TrAlc and NonTrAlc groups. To evaluate neurological status, the Glasgow Coma State (GCS) was used. In addition, we considered demographic and clinical variables including age, sex, comorbidities, medications, and the need for analgosedation, intubation or craniotomy, as well as the use of tranexamic acid or catecholamines.

## 3. Sample Processing and Protein Measurement

Blood samples were collected in biochemical tubes without anti-coagulants, and 10 min after collection, samples were centrifuged for 20 min at 3000 revolutions per minute (centrifuge MPW 352 R). Next, 1 mL of serum was transferred to 1 mL Eppendorf tubes and frozen to −20 °C. Samples are durable for up to 3 months at this temperature. Prior to analysis, tubes were thawed and brought to a temperature of 20–25 °C. Samples with visible turbidity were re-centrifuged.

Serum concentrations of S100B were determined by the electrochemiluminescence immunoassay (ECLIA) method on the cobas 6000 device calibrated with S100 CalSet. The Roche Elecsys^®^ S100 kit (Roche, Germany) with detection limits between 0.005 and 39 μg/L was used according to the manufacturer’s instructions. The cut-off value used was >0.1 μg/L. PerciControl Universal (PCU)1 and PCU2 were used for controlling the accuracy of measurements.

## 4. Statistical Analysis

The obtained data were analyzed using the Statistica 12 statistical package.

The Shapiro–Wilk test was used to examine the normal distribution of variables measurable with interval or ratio scales. The assessment of the equality of variances was based on Levene’s test. When the distribution of variables did not meet the assumptions of normal distribution, the non-parametric Mann–Whitney U test (for two analyzed groups) or the Kruskal–Wallis test (for three analyzed groups) were performed. The relationships between variables with nominal scales were estimated with the Chi^2^ test. Because multiple comparisons were conducted, we applied Bonferroni correction, dividing the assumed significance level of 0.05 by the number of comparisons. Hence, a statistically significant *p*-value was 0.0083.

## 5. Results

Patient management on admission.

Out of 107 patients (test group including TrAlc and NonTrAlc patients), 13 needed analgosedation and 12 patients required intubation. In one of the four patients with CT-confirmed intracranial hematoma, craniotomy was performed, and three of them died (on the 1st, 2nd, and 6th day, respectively). Only one patient was admitted to the Intensive Care Unit (ICU). A total of seven patients with TBI who were included in this study died. Abnormalities in CT were found in 10 patients in the TrAlc group and 7 patients in the NonTrAlc group. Patient characteristics are summarized in Table 1.

In the test group of *n* = 107 (TrAlc + NonTrAlc), 53 patients had other injuries besides TBI. The two highest levels of S100B in the entire analysis were found in patients with additional injuries, with the highest result of 32.46 μg/L in a TBI patient with spleen rupture and the second, 8.35 μg/L, in a TBI patient with bone fractures.

We analyzed the use of anticoagulants in the test group: four patients had been taking vitamin K antagonists (VKAs) and six patients had been receiving oral anticoagulants (NOACs) prior to admission to the ED. In only one patient treated with VKAs was a subdural hematoma diagnosed based on the CT scan, and the S100B level was 0.585 μg/L. In other patients in this group, no signs of TBI were found.

**Figure 1:**
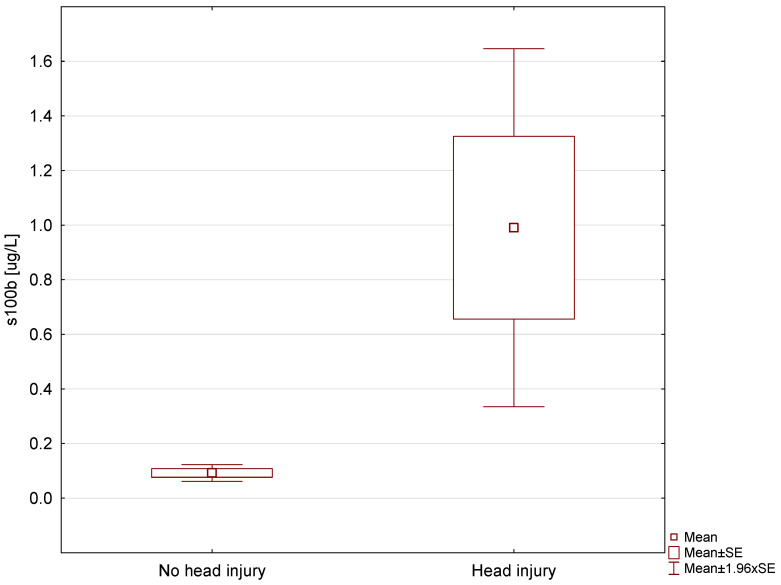
Comparison of S100B protein level between the control group and patients after head trauma.

The comparison of S100B between the control group and patients after head trauma (TrAlc + NonTrAlc) revealed that the serum marker level was higher in the latter group (0.092 vs. 0.99 μg/L), and the difference was statistically significant (*p* < 0.001) (Figure 1). However, 36 out of 107 patients with a history of injury had a lower S100B protein level than the average level in the control group.

TrAlc and NonTrAlc groups were further analyzed based on the occurrence of abnormalities revealed in CT. CT-positive groups (patients showing abnormalities in CT scans) were separately compared to the control group.

In both groups, TrAlc CT-positive and NonTrAlc CT-positive, the S100B level was significantly higher in comparison to the control group. A plasma S100B protein concentration of 1.84 μg/L (*p* < 0.00008) in the TrAlc CT-positive group and 7.592 μg/L (*p* < 0.000003) in the NonTrAlc CT-positive group as compared to control (0.092 μg/L) was observed.

The comparison of mean S100B levels in the TrAlc group with a positive CT scan versus a negative CT scan revealed a significantly higher level in the first group (1.836 μg/L vs. 0.395 μg/L; *p* = 0.0062). In the NonTrAlc group, differences were even greater. Among CT-positive patients, the mean S100B level was 7.592 μg/L, and among CT-negative patients, it was 0.373 μg/L (*p* = 0.000067).

**Figure 2:**
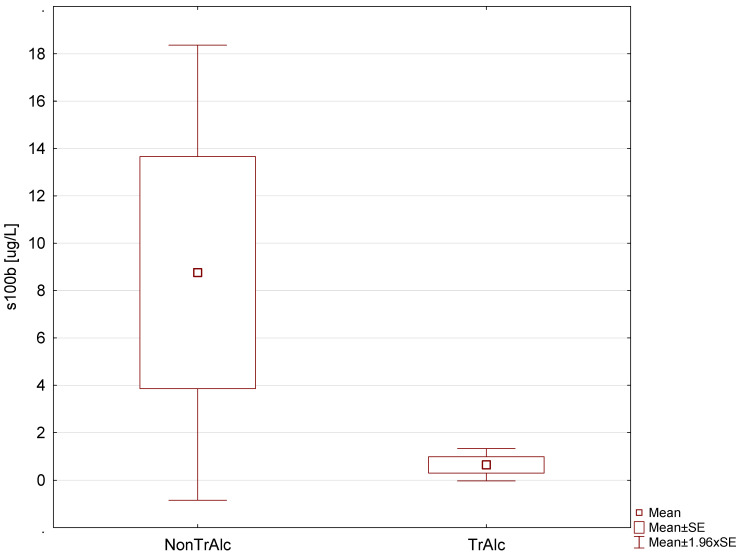
Comparison of S100B level between the NonTrAlc group and the TrAlc group (pathology in CT confirmed).

To evaluate differences between patients after alcohol intoxication and those with no intoxication, an analysis of patients with confirmed pathology in CT in NonTrAlc and TrAlc groups was performed. It revealed a higher S100B serum level in the NonTrAlc group (7.59 μg/L vs. 1.83 μg/L, Figure 2). Although the increase in the protein levels in the NonTrAlc group was over 4-fold higher than in the TrAlc group, the difference was not statistically significant (*p* = 0.088).

## 6. Discussion

S100B is a calcium-binding protein found in astroglia and other glial cells, including Schwann cells, ependymal cells, and oligodendrocytes in the CNS. It has also been found in definite neuron subpopulations [15,16,17]. The less significant fraction of S100B, referred to as an extracranial fraction, is present in striated muscle cells, heart, kidneys, and malignant melanoma cells [18,19]. The presence of S100B in tissues other than the brain tissue may contribute to the test’s low specificity in confirming TBI [20]. It is possible to measure the level of the S100B protein in the cerebrospinal fluid, and, according to Mokuno et al., it can be a promising tool for predicting the recovery of patients with neurological diseases such as Guillain–Barré syndrome [21].

**Figure 3:**
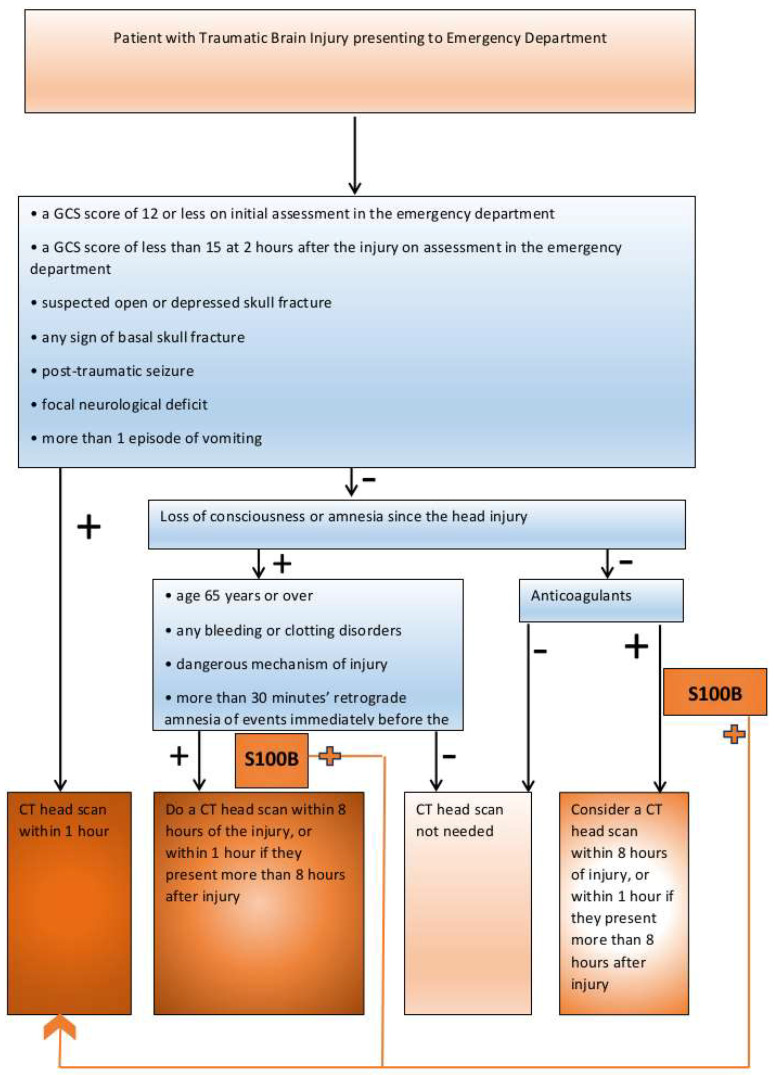
Schematic for use of serum S100B protein levels in TBI diagnosis based on NICE guidelines.

Our results indicate, with high statistical significance, that S100B protein level is much higher in patients with TBI with or without alcohol intoxication as compared to the controls. Since S100B cannot be an alternative for CT, we hope the establishment of S100B as a TBI biomarker helps in the decision making process in regard to the patients who require, e.g., a delayed CT scan [22] or no CT scan in case of patients with no loss of consciousness and fulfilling other exclusion criteria as are depicted in the algorithm below [Figure 3]. However, this approach needs to be further verified in a large group of patients.

An elevated S100B level is not specific to TBI, and it can occur in many pathologies of the CNS, such as neurogenerative diseases and congenital disorders [15,21]. Also, S100B levels can be increased due to its extracranial fraction, especially because of its presence in adipose and muscle tissues, in patients with trauma in other body regions than the head [19]. The impact of alcohol intoxication on the S100B serum level is unclear [16,23,24]. Chronic alcohol dependence can lead to higher S100B protein serum levels, and this is correlated with the amount of alcohol consumption [25]. According to Brin et al., an elevated serum level can also be an effect of acute intoxication in groups with head trauma but also among those with no trauma at all [26]. However, according to other researchers, acute alcohol intoxication should not affect the S100B serum level [16,24,27]. In a recent study, involving healthy young individuals, moderate alcohol intoxication had no effect on serum S100B levels [28]. In our study, as compared to the control group, patients with TBI had a significantly higher S100B level. Moreover, in both the TrAlc and NonTrAlc groups, the mean S100B level was higher in patients with abnormalities identified in CT, and the difference was statistically significant. In our study, we observed a decreased serum s100B in TrAlc patients (Figure 2). However, although concentrations of S100B differed between alcohol-intoxicated patients and those with no alcohol consumption, these differences were not statistically significant. Our results are consistent with the previous report where intoxicated patients with definitive TBI had lower S100B levels as compared to the sober patients [23]. This is an interesting observation, and since patients under the influence of alcohol are a large group of individuals admitted to EDs owing to head injuries, more research is necessary to determine the relationship between alcohol intake and the S100B protein level. In our opinion, this thread in the discussion is perhaps the most important for the future of assessing the usefulness of S100B protein in patients with TBI and alcohol intoxication. Its confirmation in a study with a larger sample size, or finding a cut-off point with the concentration of alcohol in the patient’s blood, may influence the recommendations regarding the use of S100B protein assays. We are planning such a study in a larger group of patients.

Despite its limitations, Scandinavian Neurotrauma Committee Guidelines included testing the S100B protein level as an addition to the clinical evaluation in patients with head trauma [27]. Data analysis suggested that incorporating this biomarker into the diagnostic algorithm enables practitioners to discharge a large proportion of patients after mild head trauma without performing CT, which can be cost-saving [27,29,30].

Establishing a TBI biomarker, potentially the S100B protein, could possibly decrease the number of unnecessary CT scans performed for patients after head trauma and economize hospital costs [31]. Our study does not include a cost–effectiveness analysis, but each biochemical test performed serially is cheaper than an imaging test. The economic aspect requires further evaluation, but the results of this study give hope for its positive development. According to Ruane et al., use of biomarkers lowers hospital costs in two situations: in medical institutions where the proportion of mild TBI patients being scanned exceeds 78% or when obtaining final CT scan results requires significantly more time than the wait for blood test results [30]. However, using the S100B protein as a pre-CT screen in other situations than those previously mentioned does not lower hospital costs due to its low specificity [4].

Another important limitation of the S100B protein as a biomarker is its changes in serum levels over time [32]. Its concentration is highest directly after head trauma and lowers over time [12]. The most significant decline in its serum level occurs 24–48 h after trauma. It returns to the normal ranges within 72 h following trauma. Therefore, when treating patients with an unknown time of trauma, the S100B test may not be useful.

The available literature provides strong evidence of an association between S100B serum levels and the probability of detecting abnormalities in CT scans after head trauma [11,33]. Therefore, it is possible to include this biomarker in already existing diagnostic algorithms, thus reducing the number of unnecessarily performed CT scans. Subsequent analyses will allow researchers to evaluate the effectiveness of this parameter. The use of biomarkers can save time and reduce diagnostic costs. However, establishing the S100B protein as a new TBI biomarker has potential limitations. First, its specificity is low [34]. Another problem is the non-apparent effect of alcohol intoxication, which is likely to influence the S100B serum level.

When treating a specific group of patients, it is not easy to decide whether diagnostic imaging should be performed. This includes cases of elderly people after falls and pregnant women. With regard to pregnant women, many guidelines indicate the assessment of maternal benefit and fetal risk. As radiation doses raise concerns, in the future, biomarkers in diagnosing mild head trauma may be considered, especially in medical centers with poor availability of MR. Patients who cannot have a CT scan without having anesthesia, for example, children and those with mental disorders, would also benefit from including biomarkers in the diagnostic process, which could help prevent unnecessary CT scans and anesthesia.

We propose a criterion that utilizes serum S100B analysis in the TBI diagnostic process based on the NICE guidelines (Figure 3).

In clinical situations, when, according to the newest NICE guidelines, a CT scan is advised but must be postponed, we suggest the S100B protein be included in the diagnostic process while waiting for imaging diagnosing [9]. The relevant group of patients can be described as follows: patients without loss of consciousness, without risk factors for severe head injury, 65 or older, with bleeding or clotting disorder, dangerous mechanism of injury, more than 30 minutes’ retrograde amnesia, or taking anticoagulant or antiplatelets. A high S100B level could indicate oligosymptomatic traumatic brain injury and, therefore, speed up the diagnostic process by performing CT immediately. We also strongly encourage considering testing S100B levels in the group of patients mentioned earlier, in whom a CT scan is essentially performed under general anesthesia. In many clinical cases, this may fill the gap in the diagnostic process.

## 7. Conclusions

The S100B protein is a promising diagnostic tool. There is a need for larger multicenter studies to establish its integration into clinical algorithms and therefore improvement of the patient care in EDs as regards radiation risks, costs, and time. Until this procedure becomes a standard, the issue of alcohol (a potential confounding factor) must be solved. Although results are promising, this analysis has limitations due to the relatively small study group. Therefore, to confirm the obtained results, more research involving a more extensive group of patients should be performed.

## Figures and Tables

**Table 1:** Characteristics of patients included in this study.

		All (*n* = 136)	NonTrAlc (*n* = 58)	TrAlc (*n* = 49)	Control (*n* = 29)	*p*
Percentage (*n*)		100% (136)	42.65% (58)	36.03% (49)	21.32% (29)	
Sex F [% (*n*)]		32.09% (43)	50.00% (29)	6.12% (3)	40.74% (11)	<0.001
Age (X_mean_/SD)		50.99/21.40	55.84/23.41	44.86/16.06	51.67 ± 23.26	0.038
No comorbidities		44.70% (59)	42.86% (24)	46.94% (23)	44.44% (12)	0.915
Comorbidities	Alcohol addiction syndrome	43.42% (33)	15.15% (5)	100% (26)	11.76% (2)	<0.001
	Cardiovascular	44.74% (34)	60.61% (20)	11.54% (3)	64.71% (11)	<0.001
	Metabolic	19.74% (15)	27.27% (9)	7.69% (2)	23.53% (4)	0.181
	Nephrological	4.00% (3)	6.25% (2)	0.00% (0)	5.88% (1)	0.555
	Oncological	5.33% (4)	9.38% (3)	0.00% (0)	5.88% (1)	0.407
	Coagulation disorders	12.00% (9)	21.88% (7)	0.00% (0)	11.76% (2)	0.062
	Neurological/psychiatric	10.67% (8)	15.63% (5)	0.00% (0)	17.65% (3)	0.122
	Pulmonary	7.89% (6)	12.50% (4)	3.70% (1)	5.88% (1)	0.537

## Data Availability

All data are included in the study.
